# Citric Acid Catalyst-Assisted Bioactive Glass with Hydrogen Peroxide for *In Vitro* Bioactivity and Biodegradability Using Sol-Gel Method

**DOI:** 10.1155/2023/9911205

**Published:** 2023-10-27

**Authors:** Tsion Chuni Aklilu, Bethelhem Gashaw Ewnete, Kena Dachasa, Kanate Sanbaba, Demeke Tesfaye, Tadele Hunde Wondimu, Jung Yong Kim, Ketema Tafess Tulu, Shimelis Lemma, Balisa Mosisa Ejeta, Fetene Fufa Bakare

**Affiliations:** ^1^Department of Materials Science and Engineering, Adama Science and Technology University, P.O. Box 1888, Adama, Ethiopia; ^2^Center of Advanced Materials Science and Engineering, Adama Science and Technology University, P.O. Box 1888, Adama, Ethiopia; ^3^Institute of Pharmaceutical Sciences, Adama Science and Technology University, P.O. Box 1888, Adama, Ethiopia; ^4^Department of Applied Biology, School of Applied Natural Science, Adama Science and Technology University, P.O. Box 1888, Adama, Ethiopia; ^5^Bio and Emerging Institute of Technology, P.O. Box 5954, Addis Ababa, Ethiopia

## Abstract

In this study, carbon-free and completely soluble hydrogen peroxide (H_2_O_2_) was utilized in place of conventional surfactants as a pore-forming agent. Citric acid was also used in low concentration for the hydrolysis reaction. A sol-gel method was used to prepare bioactive glass (BG) specimens of H_2_O_2_-untreated BG, 1M, 2M, and 3M H_2_O_2_-treated BGs. X-ray diffraction (XRD), scanning electron microscopy (SEM), Fourier-transform infrared spectroscopy (FTIR), energy-dispersive spectroscopy (EDS), and nitrogen adsorption/desorption isotherm with the Brunauer–Emmett–Teller (BET) method were used for analyzing the samples' phase, surface morphology, chemical composition, constituent composition, pore size, and specific surface area respectively. In vitro bioactivity, as well as biodegradability tests, was performed on samples by immersing them in simulated body fluid (SBF) solution. According to the results, BG particles treated with 2 M H_2_O_2_ exhibited higher specific surface area (SSA), which is 189.55 cc/g, and better in vitro bioactivity and biodegradability.

## 1. Introduction

The body's musculoskeletal system is essential in that it is constantly susceptible to injury or damage by illnesses, pathologies, trauma, infections, physiological resorption, and age [1]. A lot of research has been done to find an appropriate material for these musculoskeletal injuries and illnesses. For example, the bioactive ceramics like glass-ceramics, hydroxyapatite (HAp), and bioactive glasses (BG) have been utilized, affording a particular biological reaction through the formation of chemical bonds on their intended bone-tissue surfaces [2–5]. Among these materials, BGs received great attention due to their cell proliferation, osteogenesis, and osteoproductive properties [6, 7]. Particularly, BG has been commonly used for dental and bone restoration. This is because BG is biocompatible, biodegradable, and bioactive for promoting bone healing [5, 8]. The primary inorganic constituent of bone is HAp, which has been found to chemically bond with human bones, indicating that the substance has a bioactive nature [9–11]. For a variety of purposes, degradable materials are usually preferred over durable implant materials because they can be replaced by regenerated tissues and concomitantly absorbed by the body [12, 13]. It is generally recognized that the products of BGs' ion dissolution promote angiogenesis, osteogenesis, and vascularization [14, 15].

The earliest and most thoroughly studied BG, called “45S5 Bioglass®,” is regarded as the industry standard because it can chemically adhere to bone tissue despite developing non-adherent fibrous capsules [16]. However, it was synthesized through a melt-processing requiring a relatively high melting temperature (1300–1500°C), and thus, it has no internal microporous network, resulting in a significant impact on the bioactivity of bone in later phases [17]. In most situations, an increase in SiO_2_ in melted BGs will lead to a decrease in the glass' bioavailability. Even after being subjected to simulated bodily fluid (SBF) for several weeks, the BGs that contain more than 60% SiO_2_ are unable to develop a carbonated hydroxyapatite layer and do not bind to soft or bone tissues [18].

Thus, the BGs were prepared by another processing method like the sol-gel technique, which allows a greater surface area through a porous microstructure, enabling a wider range of applications [15, 19, 20]. In addition, they exhibited a relatively higher rate of bone bonding along with improved resorption and degradation characteristics [21]. Adjusting the processing parameters can cause significant changes in the characteristics of the product, including its morphology and composition [15]. Then, different structure-directing agents have been investigated in this sol-gel processing for inducing desirable porous bioactive glass and enhancing the bioactivity and biodegradability properties [22]. The porous structure in BGs enables the movement of cells, mass transfer, food supply, oxygen diffusion, and the removal of metabolic wastes, all of which are essential for the growth of new tissues [20]. Moreover, these BG materials display excellent surface textural properties and well-defined mesoporous structure with adjustable pore size (e.g., large pore volume and high surface area) [23].

Cetyltrimethylammonium bromide (CTAB), poly(ethylene glycol) (PEG), polyurethane (PU), and Pluronics (i.e., synthetic block copolymers such as P123: EO20-PO70-EO20 and F127: EO106-PO70-EO106) are the typical surfactants used as pore-forming agents for synthesizing porous BGs [24–27]. However, the use of surfactants could lead to problems such as micelle aggregation and carbon contamination [28]. The final specimen's pores and surface area will be reduced as a result of the carbon contamination as well as micelle aggregation, leading to particles with wrinkled surfaces [29]. Because these inhibit HAp from forming a proper interfacial linkage, it results in a reduction in the bioactivity and biodegradability properties [28, 30]. It was reported that a high-purity BG with a high specific surface area can be manufactured if hydrogen peroxide (H_2_O_2_) are employed as a pore-forming agent [30]. Using hydrogen peroxide (H_2_O_2_) as a novel pore-forming agent and BG with high SSA resulting from its decomposition behavior at 180°C (2H_2_O_2_ ⟶ 2H_2_O + O_2_) produces significant amounts of oxygen gas to build the pore structures [28, 30]. Importantly, H_2_O_2_ has no carboxyl groups and can be entirely soluble in water, allowing no carbon contamination as well as aggregation effects. Hence, H_2_O_2_ can substitute traditional surfactants in the role of pore formation.

The acidic or basic conditions used for the catalysis of hydrolysis during the sol-gel technique have a considerable impact on the properties of BG [31]. Inorganic acid catalysts and basic catalysts are used at higher concentration in sol-gel process. Therefore, it is appropriate to look for ways to make the procedure greener while avoiding the typical high-temperature or extreme pH conditions found in the numerous previous protocols [32]. For this reason, the biological hydroxyl-carboxyl acid catalysts such as acetic acid, lactic acid, and citric acid have been utilized in a lower concentration, resulting in enhanced bioactivity and biodegradability [20]. Citric acid contains distinctive chemical structures affecting the structural characteristics of BGs such as the particle size and porosity when it was employed for the sol-gel procedure [33]. In comparison to other acids such as nitric and hydrochloric acids requiring a higher concentration, citric acid has a great catalysis activity at lower concentration [34, 35]. On the other hand, pH levels of acids cause more roughness and porosity in case of organic acids than inorganic ones, so that nucleation of HAp takes place in more spots for organic acid-synthesized BGs [20]. Hence, in this work, we investigated the impacts of the sol–gel processing technique with citric acid and hydrogen peroxide on BGs' bioactivity and biodegradability properties.

## 2. Materials and Methods

### 2.1. Synthesis of BG Powders

In this study, BG powder with a 58S composition (60mol% SiO_2_, 36mol% CaO, and 4mol% P_2_O_5_) was synthesized using the sol-gel synthesis method. Precursors used as Si, Ca, and P sources include tetraethyl orthosilicate (TEOS, Si(OC_2_H_5_)_4_), calcium nitrate tetrahydrate (CN, Ca(NO_3_)_2_.4H_2_O), and phosphoric acid (H_3_PO_4_), respectively. 0.5 mM citric acid was added in 50 mL distilled water and ethanol alcohol (1 : 1) solvents and stirred for 15 minutes at room temperature. The two solvents were used together because of solubility differences between the precursors. TEOS is highly soluble in ethanol, whereas CN and H_3_PO_4_ are soluble in water. Additionally, the citric acid solution is used to catalyze the hydrolysis of silicon alkoxide (TEOS) and plays a great role in the morphology of bioactive glass that enhances the bioactivity and biodegradability properties. When ethanol and water are mixed together, they form an alcohol-water mixture which can effectively dissolve organic compounds including citric acid. The solubility of citric acid or other solutes in the alcohol-water solution depends on the proportion of these solvents. Using equal amounts of these solvents promote enhanced solubility and versatile solvent system which allow solute dissolution while maintaining polar properties of water and organic properties of ethanol. Then, TEOS, phosphoric acid, calcium nitrate tetrahydrate, and hydrogen peroxide were added in to the solution sequentially. The precursor solution was stirred on a hot plate with a magnetic stirrer for 2 hours at room temperature in order to create a homogeneous solution. The prepared sol was aged and gelled for 24 hours at 60°C. Then, the obtained gel was dried at 100°C for 3 hr to remove solvents. The dried specimens were calcined for 2 hr at 600°C to remove the remaining precursor residues and solvents. The sample without using any H_2_O_2_ was denoted as BG0. 1M, 2M, and 3M H_2_O_2_-treated BG were denoted as BG1, BG2, and BG3, respectively.

### 2.2. Characterization

After sample preparation, analyses of phase, surface morphology, chemical composition, constituent composition, and specific surface area were performed. The X-ray diffraction (XRD), scanning electron microscopy (SEM), Fourier-transform infrared microscopy (FTIR), energy-dispersive x-ray spectroscopy (EDS), and Brunauer–Emmett–Teller (BET) surface analysis were used to obtain details of phase composition, surface morphologies of the specimens, chemical identification, identify the constituent composition, and the specific surface area of each specimen, respectively.

### 2.3. *In Vitro* Bioactivity Test

*In vitro* bioactivity studies have previously used Kokubo's simulated bodily fluid (SBF) test solution [36]. All BG specimens were soaked into a simulated body fluid (SBF) having an ionic content comparable with typical human plasma, to test *in vitro* bioactivity of all BGs. Each of the specimens was then submerged in SBF and kept in a water bath that was maintained at a constant 37°C for 3 days. HAp layer formation on soaked samples was characterized by XRD and FTIR for phase and composition analysis after rinsing the soaked samples with distilled water three times and dried at 80°C for 3 days. The Debye-Scherer equation (equation (1)) is used to calculate each of the specimen's bioactivity after using the peak area analysis of HAp to estimate the crystallite size. A biodegradability test was also done measuring the weight loss of pellet specimens in SBF solution after rinsing soaked samples.

(1) $$\begin{array}{c} D=\frac{K\lambda }{\beta \;\cos\;\theta }, \end{array}$$

where *θ* is the diffraction angle, *β* is the full width at half maximum (FWHM) of the diffraction peak, *λ* is the X-ray wavelength (1.54178Å), *D* is the crystallite size, and *K* (=0.94) is the Scherer's constant.

### 2.4. *In Vitro* Biodegradability Test

BG0, BG1, BG2, and BG3 specimens in pellet form were examined for in vitro biodegradability in SBF solution. For each specimen, three BG pellets were utilized to calculate the weight loss's average and standard deviation. The ratio of 1 gm to 10 mL of SBF was used to submerge the measured and produced BG powder pellets. Every day, the solutions were discarded, and the pellets were dried and weighed once more. Every day, the entire assessment was repeated for 30 days, replacing the SBF.

## 3. Results and Discussion

**Figure 1:**
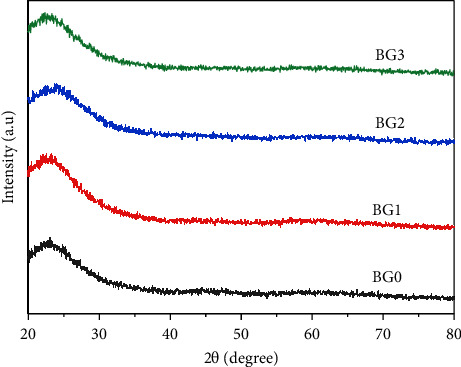
XRD patterns of BG0, BG1, BG2, and BG3 powders before soaking in SBF.

Figure 1 shows the XRD patterns of BG0, BG1, BG2, and BG3 powders. The broad peak observed from 2*θ* = 20° to 30° suggests that the BG specimens were effectively synthesized without a sharp X-ray diffraction peak, indicating an amorphous phase, i.e., the absence of ordered crystal structure.

**Figure 2:**
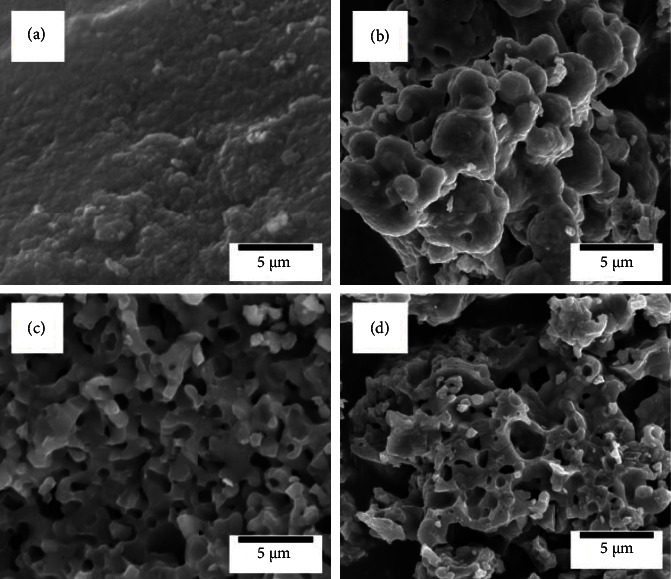
SEM images of (a) BG0 (b) BG1, (c) BG2, and (d) BG3 before soaking in SBF.

Figure 2 shows the surface morphologies of BG0, BG1, BG2, and BG3 before soaking in SBF. In Figure 2(a), the BG0 particles were observed to have rough surface morphology. The BG particles that had been treated with H_2_O_2_ exhibited porous morphologies. Figure 2(b) illustrates that 1M H_2_O_2_-treated BG particles exhibited aggregation and some porous particles. For the BG2 particles with 2M H_2_O_2_, more uniform and narrower pore distributions are seen from Figure 2(c). On the other hand, in case of 3M H_2_O_2_, relatively larger holes with irregular distributions were seen as illustrated in Figure 2(d). This morphology might be caused by H_2_O_2_ gathering into the larger clusters, creating wider pores.

**Figure 3:**
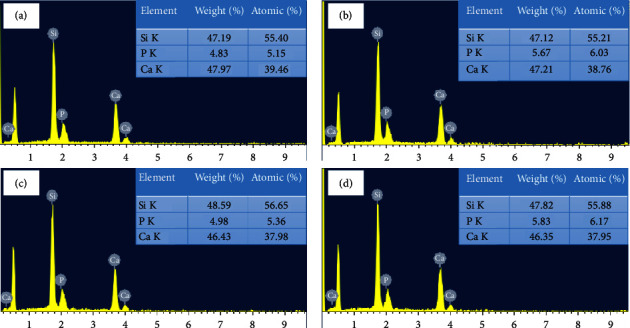
EDS spectra of (a) BG0, (b) BG1, (c) BG2, and (d) BG3 powders.

The EDS spectra in Figure 3 demonstrate that all BG0, BG1, BG2, and BG3 powders contain silicon, calcium, and phosphor elements, which are the components of all silicate BGs [37]. Additionally, it shows any residual oxygen and other components successfully removed throughout the heating process. Since H_2_O_2_ induces the formation of oxygen for pore formation, its full elimination shows the successful pore formation process enhancing the specific surface area of the particles.

**Figure 4:**
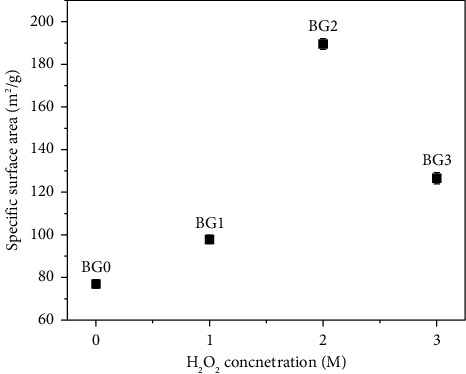
Specific surface areas of BG0, BG1, BG2, and BG3 powders.

Specific surface areas and pore size of all BG specimens were analyzed using the BET measurements as shown in Table 1. The specific surface areas obtained from the BET measurements for BG0, BG1, BG2, and BG3 powders are 77.02 ± 1.02, 97.877 ± 0.95, 189.55 ± 1.73, and 126.56 ± 1.66 m^2^/g, respectively. According to the BET results, which are shown in Figure 4, as we increased the H_2_O_2_ concentration from 0 to 2M, the specific surface area were increased. However, in 3M H_2_O_2_-treated BG powder, the specific surface area were decreased due to the H_2_O_2_ molecules being accumulated into larger clusters, leading to larger pores.

On the other hand pore size of BG0, BG1, BG2, and BG3 powders were 1.65 ± 0.05, 1.63 ± 0.071, 1.52 ± 0.053, and 2.51 ± 0.046 nm, respectively, as shown on Table 1. 3M H_2_O_2_ (BG3) shows the highest pore size due to the highest concentration of the H_2_O_2_ molecules being aggregated into larger clusters that created larger pores [30].

Table 1 provides an overview of all the pore size and specific surface areas of the BG specimens. The data were presented as an average standard deviation (SD) for *n* = 3 in which every sample reveals *p* <  0.05 [38–40]. The variance in the *p* value shows there is an important distinction between the BG specimens.

**Figure 5:**
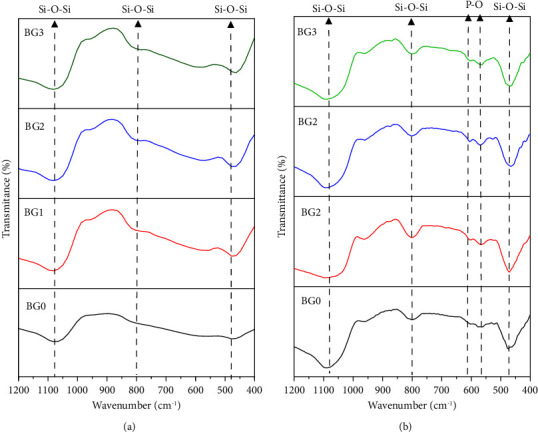
FTIR spectra of BG0, BG1, BG2, and BG3 powders (a) before soaking in SBF and (b) after soaking in SBF for 3 days.

FTIR spectra are shown in Figure 5, for the bioactivity tests of BG0, BG1, BG2, and BG3 specimens.  Figure 5(a) illustrates that the spectra for the as prepared samples at 1090 cm^−1^, 800 cm^−1^, and 482 cm^−1^ exhibit only Si-O-Si bands. After BG powders were soaked in SBF for 3 days, all specimens showed a new peak that occurred at 566 cm^−1^ and was assigned to the P-O band [41], as shown in Figure 5(b).

**Figure 6:**
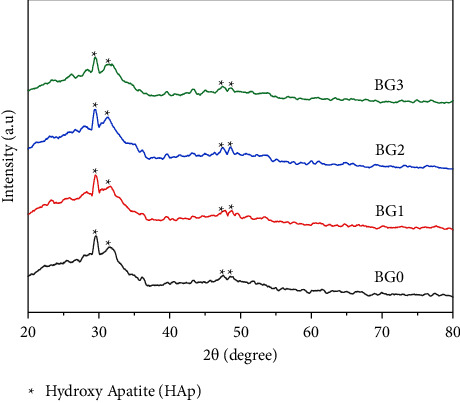
XRD patterns of BG0, BG1, BG2, and BG3 powders after soaking in SBF for 3 days.

The XRD pattern of BG0, BG1, BG2, and BG3 powders that were soaked in SBF for 3 days is shown in Figure 6. Diffraction peaks were observed at 29.46°, 31.38°, and weak peaks were observed at 47.68° and 48.86° which correspond to the development of hydroxyapatite layer [42, 43]. The crystallite size was calculated from the XRD pattern in order to determine the bioactivity of each specimen. The bioactivity value increases as crystallite size for HAp formation increases. Additionally, the XRD data demonstrate that BG powder treated with 2M H_2_O_2_ produces superior crystallite sizes than the other BG specimens, which suggests a higher formation of HAp and higher bioactivity.

**Figure 7:**
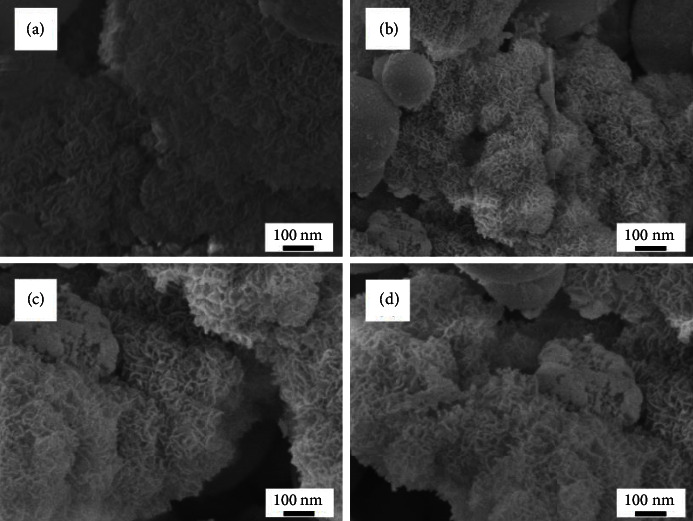
SEM images of BG0, BG1, BG2, and BG3 powders after soaking in SBF for 3 days.

As shown on Figure 7, *in vitro* bioactivity analyses were obtained from SEM images after soaking in SBF for 3 days. They illustrate that smaller crystallites are formed and distributed on the surface of the BG particles which demonstrate the formation of HAp layers on BG particles.

**Figure 8:**
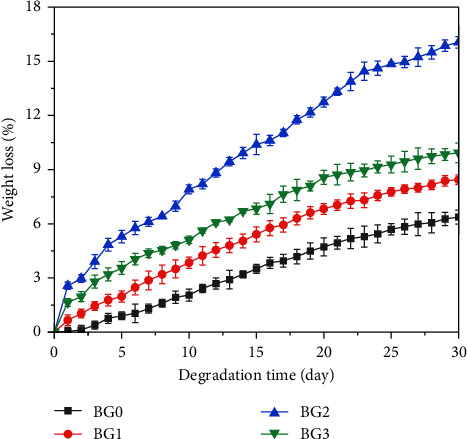
Weight loss of BG0, BG1, BG2, and BG3 after soaking in SBF solution for 30 days.

Figure 8 shows the results of a 30 days on the biodegradation properties of BG pellets treated with 1M, 2M, and 3M H_2_O_2_ in SBF solution. The graph demonstrates that the weight loss of the BG0, BG1, BG2, and BG3 samples was 6.37 ± 0.386%, 8.43 ± 0.271%, 16.04 ± 0.315%, and 9.93 ± 0.540%, respectively, after being soaked for 30 days in SBF solution. In addition, the degradation rate of BG0, BG1, BG2, and BG3 specimens were 0.21 ± 0.013%, 0.28 ± 0.009%, 0.53 ± 0.011%, and 0.33 ± 0.018%, respectively. The results shown that 2M H_2_O_2_-treated BG had the highest weight loss.

**Figure 9:**
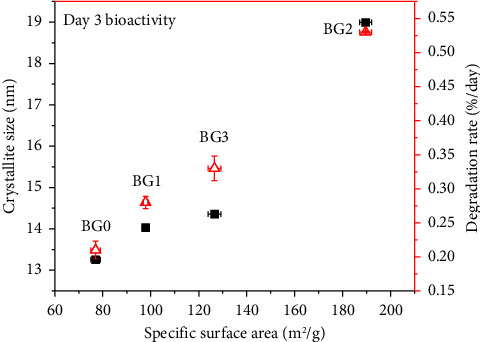
Correlation between bioactivity and biodegradability as a function of specific surface area.

According to Figure 9, bioactivity and biodegradability were correlated with specific surface area; as a result, the BG's better bioactivity and biodegradability are related to the powders with higher specific surface area [41]. Specific surface area enhances with H_2_O_2_ concentration from which the bioactivity and biodegradation of BGs are determined. However, for 3M H_2_O_2_-treated BG, specific surface area shows decrement. Thus, it leads to reduce the bioactivity as well as biodegradability of the specimens.

**Figure 10:**
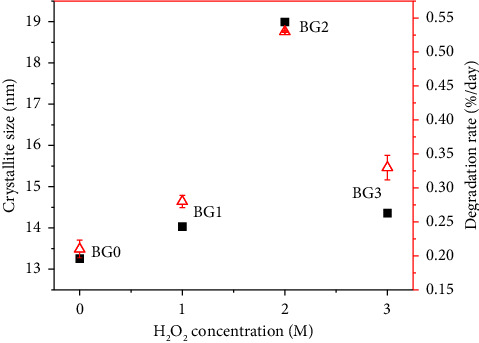
Correlation between bioactivity and degradation rate as a function of H_2_O_2_ concentration.

Because of its rapid ability to produce oxygen through its 2H_2_O_2_ ⟶ 2H_2_O + O_2_ decomposition characteristic, BG treated with H_2_O_2_ has a higher specific surface area [30]. Therefore, an enormous amount of oxygen gas would be produced to form the pore structures. However, bioactivity and biodegradability decreases for higher concentrations of H_2_O_2_. This is mainly due to H_2_O_2_ molecules may have aggregated into larger clusters which will hinder the formation of HAp as well as the degradation of the BG specimens [30].  Figure 10 shows bioactivity as a function of H_2_O_2_ concentration. It demonstrates that H_2_O_2_-treated BG specimens show better bioactivity than the untreated one. As the H_2_O_2_ concentration increases up to 2M, bioactivity also increases. But raising the concentration to 3M results the bioactivity to decrease.

## 4. Conclusion

In this study, the bioactive glass (BG0, BG1, BG2, and BG3) powders were successfully synthesized using the sol-gel method. The results of this study generally indicate that utilizing H_2_O_2_ as a pore-forming agent enhance BG's specific surface area, bioactivity, and biodegradability. The formation of BG particles with a high specific surface area has been made possible by the high solubility and rapid oxygen producing capabilities of H_2_O_2_ through its 2H_2_O_2_ ⟶ 2H_2_O + O_2_ decomposition. According to the BET analysis, the BG particles with high specific surface area were obtained for 2M H_2_O_2_ concentrations. The *in vitro* bioactivity test was done by SEM, XRD, as well as FTIR analysis and reveals crystal phase (HAp) formation. Thus, it would enhance bioactivity and biodegradability properties of BG.

## Figures and Tables

**Table 1:** Specific surface area and pore size of BG0, BG1, BG2, and BG3 powders.

Samples	Specific surface area	*p* value	Pore size (nm)	*p* value
BG0	77.02 ± 2.02	0.044	1.654 ± 0.05	0.024
BG1	97.87 ± 1.95	0.036	1.650 ± 0.071	0.009
BG2	189.55 ± 1.73	0.033	1.520 ± 0.053	0.011
BG3	126.56 ± 2.66	0.032	2.507 ± 0.046	0.007

## Data Availability

The data used to support the findings of this study are included in the article.
